# A computational model for functional mapping of genes that regulate intra-cellular circadian rhythms

**DOI:** 10.1186/1742-4682-4-5

**Published:** 2007-01-30

**Authors:** Tian Liu, Xueli Liu, Yunmei Chen, Rongling Wu

**Affiliations:** 1 Department of Statistics, University of Florida, Gainesville, FL 32611, USA; 2 Department of Mathematics, University of Florida, Gainesville, FL 32611, USA

## Abstract

**Background:**

Genes that control circadian rhythms in organisms have been recognized, but have been difficult to detect because circadian behavior comprises periodically dynamic traits and is sensitive to environmental changes.

**Method:**

We present a statistical model for mapping and characterizing specific genes or quantitative trait loci (QTL) that affect variations in rhythmic responses. This model integrates a system of differential equations into the framework for functional mapping, allowing hypotheses about the interplay between genetic actions and periodic rhythms to be tested. A simulation approach based on sustained circadian oscillations of the clock proteins and their mRNAs has been designed to test the statistical properties of the model.

**Conclusion:**

The model has significant implications for probing the molecular genetic mechanism of rhythmic oscillations through the detection of the clock QTL throughout the genome.

## Background

Rhythmic phenomena are considered to involve a mechanism, ubiquitous among organisms populating the earth, for responding to daily and seasonal changes resulting from the planet's rotation and its orbit around the sun [1]. All these periodic responses are recorded in a circadian clock that allows the organism to anticipate rhythmic changes in the environment, thus equipping it with regulatory and adaptive machinery [2]. It is well recognized that circadian rhythms operate at all levels of biological organization, approximating a twenty-four hour period, or more accurately, the alternation between day and night [3]. Although there is a widely accepted view that the normal functions of biological processes are strongly correlated with the genes that control them, the detailed genetic mechanisms by which circadian behavior is generated and mediated are poorly understood [4].

Several studies have identified various so-called circadian clock genes and clock-controlled transcription factors through mutants in animal models [5,6]. These genes have implications for clinical trials: their identification holds great promise for determining optimal times for drug administration based on an individual patient's genetic makeup. It has been suggested that drug administration at the appropriate body time can improve the outcome of pharmacotherapy by maximizing the potency and minimizing the toxicity of the drug [7], whereas drug administration at an inappropriate body time can induce more severe side effects [8]. In practice, body-time-dependent therapy, termed chronotherapy [9], can be optimized via the genes that control expression of the patient's physiological variables during the course of a day.

With the completion of the Human Genome Project, it has been possible to draw a comprehensive picture of the genetic control of the functions of the biological clock and, ultimately, to integrate genetic information into routine clinical therapies for disease treatment and prevention. To achieve this goal, there is a pressing need to develop powerful statistical and computational algorithms for detecting genes or quantitative trait loci that determine circadian rhythms as complex dynamic traits. Unlike many other traits, rhythmic oscillations are generated by complex cellular feedback processes comprising a large number of variables. For this reason, mathematical models and numerical simulations are needed to grasp the molecular mechanisms and functions of biological rhythms fully [10]. These mathematical models have proved useful for investigating the dynamic bases of physiological disorders related to perturbations of biological behavior.

In this article, we will develop a statistical model for genetic mapping of QTL that determine patterns of rhythmic responses, using random samples from a natural population. This model is implemented by the principle of functional mapping [11], a statistical framework for mapping dynamic QTL for the pattern of developmental changes, by considering systems of differential equations for biological clocks. Simulation studies have been performed to investigate the statistical properties of the model.

## Model

### Mathematical Modeling of Circadian Rhythms

**Figure 1:**
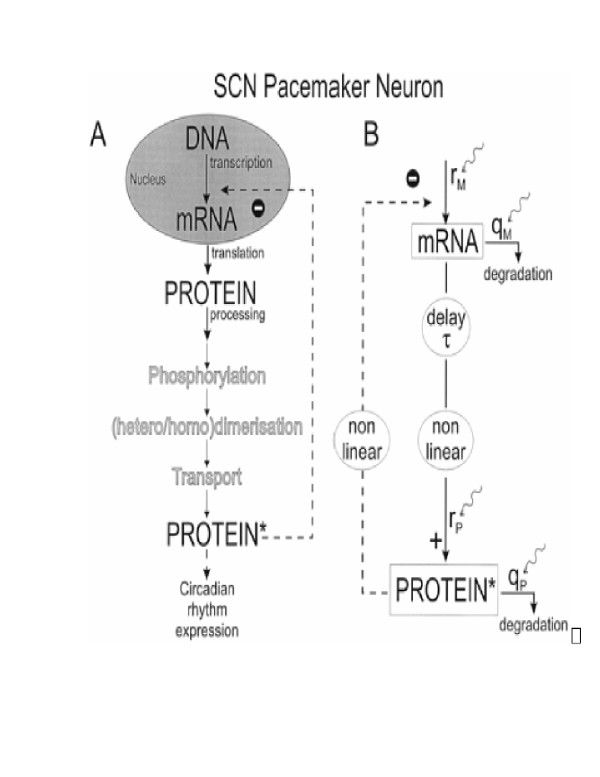
(A) Diagram of the biological elements of the protein synthesis cascade for a circadian rhythm generator. (B) Model interpretation of A showing the delay (*τ*) and nonlinearity in the protein production cascade, the nonlinear negative feedback, and mRNA and protein production (*r*_*M*_, *r*_*P*_) and degradation (*q*_*M*_, *q*_*P*_). Adapted from ref. [10].

In all organisms studied so far, circadian rhythms that allow adaptation to a periodically changing environment originate from negative autoregulation of gene expression. Scheper et al. [10] illustrated and analyzed the generation of a circadian rhythm as a process involving a reaction cascade containing a loop, as depicted in Fig. 1A. The reaction loop consists in the production of the effective protein from its mRNA and negative feedback from the effective protein on mRNA production. The protein production process involves translation and subsequent processing steps such as phosphorylation, dimerization, transport and nuclear entry. It is assumed that the protein production cascade and the negative feedback are nonlinear processes in the reaction loop (Fig. 1B), with a time delay between protein production and subsequent processing. These nonlinearities and the delay critically determine the free-running periodicity in the feedback loop.

Scheper et al. [10] proposed a system of coupled differential equations to describe the circadian behavior of the intracellular oscillator:

dMdt=rM1+(Pk)n−qMMdPdt=rPM(t−τ)m−qPP     (1)
 MathType@MTEF@5@5@+=feaafiart1ev1aaatCvAUfKttLearuWrP9MDH5MBPbIqV92AaeXatLxBI9gBaebbnrfifHhDYfgasaacH8akY=wiFfYdH8Gipec8Eeeu0xXdbba9frFj0=OqFfea0dXdd9vqai=hGuQ8kuc9pgc9s8qqaq=dirpe0xb9q8qiLsFr0=vr0=vr0dc8meaabaqaciaacaGaaeqabaqabeGadaaakeaafaqaaeGabaaabaWaaSaaaeaacqWGKbazcqWGnbqtaeaacqWGKbazcqWG0baDaaGaeyypa0ZaaSaaaeaacqWGYbGCdaWgaaWcbaGaemyta0eabeaaaOqaaiabigdaXiabgUcaRmaabmaabaWaaSaaaeaacqWGqbauaeaacqWGRbWAaaaacaGLOaGaayzkaaWaaWbaaSqabeaacqWGUbGBaaaaaOGaeyOeI0IaemyCae3aaSbaaSqaaiabd2eanbqabaGccqWGnbqtaeaadaWcaaqaaiabdsgaKjabdcfaqbqaaiabdsgaKjabdsha0baacqGH9aqpcqWGYbGCdaWgaaWcbaGaemiuaafabeaakiabd2eanjabcIcaOiabdsha0jabgkHiTGGaciab=r8a0jabcMcaPmaaCaaaleqabaGaemyBa0gaaOGaeyOeI0IaemyCae3aaSbaaSqaaiabdcfaqbqabaGccqWGqbauaaGaaCzcaiaaxMaadaqadaqaaiabigdaXaGaayjkaiaawMcaaaaa@5C91@

where *M *and *P *are, respectively, the relative concentrations of mRNA and the effective protein measured at a particular time, *r*_*M *_is the scaled mRNA production rate constant, *r*_*P *_is the protein production rate constant, *q*_*M *_and *q*_*P *_are, respectively, the mRNA and protein degradation rate constants, *n *is the Hill coefficient, *m *is the nonlinear exponent in the protein production cascade, *τ *is the total duration of protein production from mRNA, and *k *is a scaling constant.

Equation 1 constructs an unperturbed (free-running) system of the intracellular circadian rhythm generator that is defined by seven parameters, Θ_*u *_= (*n*, *m*, *τ*, *r*_*M*_, *r*_*P*_, *q*_*M*_, *q*_*P*_, *k*). The behavior of this system can be determined and predicted by changes in these parameter combinations. For a given QTL, differences in the parameter combinations among genotypes imply that this QTL is involved in the regulation of circadian rhythms. Statistical models will be developed to infer such genes from observed molecular markers such as single nucleotide polymorphisms (SNPs).

### Statistical Modeling of Functional Mapping

Suppose a random sample of size *N *is drawn from a natural human population at Hardy-Weinberg equilibrium. In this sample, multiple SNP markers are genotyped, with the aim of identifying QTL that affect circadian rhythms. The relative concentrations of mRNA (*M*) and the effective protein (*P*) are measured in each subject at a series of time points (1, ..., *T*), during a daily light-dark cycle. Thus, there are two sets of serial measurements, expressed as [*M*(1), ..., *M*(*T*)] and [*P*(1), ..., *P*(*T*)]. According to the differential functions (1), these two variables, modeled in terms of their change rates, are expressed as differences between two adjacent times, symbolized by

**y **= [*M*(2) - *M*(1),..., *M*(*T*) - *M*(*T *- 1)]

= [*y*(1),..., *y*(*T *- 1)]

for the protein change and

**z **= [*P*(2) - *P*(1),..., *P*(*T*) - *P*(*T *- 1)]

= [*z*(1),..., *z*(*T *- 1)]

for the mRNA change.

Assume that a putative QTL with alleles *A *and *a *affecting circadian rhythms is segregated in the population. The frequencies of alleles *A *and *a *are *q *and 1 - *q*, respectively. For a particular genotype *j *of this QTL *(j *= 0 for *aa*, 1 for *Aa *and 2 for *AA*), the parameters describing circadian rhythms are denoted by **Θ**_***uj ***_= (*n*_*j*_, *m*_*j*_, *τ*_*j*_, *r*_*Mj*_, *r*_*Pj*_, *q*_*Mj*_, *q*_*Pj*_, *k*_*j*_). Comparisons of these quantitative genetic parameters among the three different genotypes can determine whether and how this putative QTL affects circadian rhythms.

The time-dependent phenotypic changes in mRNA and protein traits for individual *i *measured at time *t *due to the QTL can be expressed by a bivariate linear statistical model

yi(t)=∑j=02ξijuMj(t)+eiy(t)zi(t)=∑j=02ξijuPj(t)+eiz(t)     (2)
 MathType@MTEF@5@5@+=feaafiart1ev1aaatCvAUfKttLearuWrP9MDH5MBPbIqV92AaeXatLxBI9gBaebbnrfifHhDYfgasaacH8akY=wiFfYdH8Gipec8Eeeu0xXdbba9frFj0=OqFfea0dXdd9vqai=hGuQ8kuc9pgc9s8qqaq=dirpe0xb9q8qiLsFr0=vr0=vr0dc8meaabaqaciaacaGaaeqabaqabeGadaaakeaafaqaaeGabaaabaGaemyEaK3aaSbaaSqaaiabdMgaPbqabaGccqGGOaakcqWG0baDcqGGPaqkcqGH9aqpdaaeWbqaaGGaciab=57a4naaBaaaleaacqWGPbqAcqWGQbGAaeqaaOGaemyDau3aaSbaaSqaaiabd2eanjabdQgaQbqabaGccqGGOaakcqWG0baDcqGGPaqkaSqaaiabdQgaQjabg2da9iabicdaWaqaaiabikdaYaqdcqGHris5aOGaey4kaSIaemyzau2aa0baaSqaaiabdMgaPbqaaiabdMha5baakiabcIcaOiabdsha0jabcMcaPaqaaiabdQha6naaBaaaleaacqWGPbqAaeqaaOGaeiikaGIaemiDaqNaeiykaKIaeyypa0ZaaabCaeaacqWF+oaEdaWgaaWcbaGaemyAaKMaemOAaOgabeaakiabdwha1naaBaaaleaacqWGqbaucqWGQbGAaeqaaOGaeiikaGIaemiDaqNaeiykaKcaleaacqWGQbGAcqGH9aqpcqaIWaamaeaacqaIYaGma0GaeyyeIuoakiabgUcaRiabdwgaLnaaDaaaleaacqWGPbqAaeaacqWG6bGEaaGccqGGOaakcqWG0baDcqGGPaqkaaGaaCzcaiaaxMaadaqadaqaaiabikdaYaGaayjkaiaawMcaaaaa@74CF@

where *ξ*_*ij *_is an indicator variable for the possible genotypes of the QTL for individual *i*, defined as 1 if a particular QTL genotype *j *is indicated and 0 otherwise, *u*_M*j*_(*t*) and *u*_P*j*_(*t*) are the genotypic values of the QTL for mRNA and protein changes at time *t*, respectively, which can be determined using the differential functions expressed in equation (1), and eiy
 MathType@MTEF@5@5@+=feaafiart1ev1aaatCvAUfKttLearuWrP9MDH5MBPbIqV92AaeXatLxBI9gBaebbnrfifHhDYfgasaacH8akY=wiFfYdH8Gipec8Eeeu0xXdbba9frFj0=OqFfea0dXdd9vqai=hGuQ8kuc9pgc9s8qqaq=dirpe0xb9q8qiLsFr0=vr0=vr0dc8meaabaqaciaacaGaaeqabaqabeGadaaakeaacqWGLbqzdaqhaaWcbaGaemyAaKgabaGaemyEaKhaaaaa@3102@(*t*) and eiz
 MathType@MTEF@5@5@+=feaafiart1ev1aaatCvAUfKttLearuWrP9MDH5MBPbIqV92AaeXatLxBI9gBaebbnrfifHhDYfgasaacH8akY=wiFfYdH8Gipec8Eeeu0xXdbba9frFj0=OqFfea0dXdd9vqai=hGuQ8kuc9pgc9s8qqaq=dirpe0xb9q8qiLsFr0=vr0=vr0dc8meaabaqaciaacaGaaeqabaqabeGadaaakeaacqWGLbqzdaqhaaWcbaGaemyAaKgabaGaemOEaOhaaaaa@3104@(*t*) are the residual effects in individual *i *at time *t*, including the aggregate effect of polygenes and error effects.

The dynamic features of the residual errors of these two traits can be described by the antedependence model, originally proposed by Gabriel [12] and now used to model the structure of a covariance matrix [13]. This model states that an observation at a particular time *t *depends on the previous ones, the degree of dependence decaying with time lag. Assuming the 1^st^-order structured antedependence (SAD(1)) model, the relationship between the residual errors of the two traits *y *and *z *at time *t *for individual *i *can be modeled by

eiy(t)=φzeiy(t−1)+ψzeiz(t−1)+εiy(t)eiz(t)=φzeiz(t−1)+ψzeiy(t−1)+εiz(t)     (3)
 MathType@MTEF@5@5@+=feaafiart1ev1aaatCvAUfKttLearuWrP9MDH5MBPbIqV92AaeXatLxBI9gBaebbnrfifHhDYfgasaacH8akY=wiFfYdH8Gipec8Eeeu0xXdbba9frFj0=OqFfea0dXdd9vqai=hGuQ8kuc9pgc9s8qqaq=dirpe0xb9q8qiLsFr0=vr0=vr0dc8meaabaqaciaacaGaaeqabaqabeGadaaakeaafaqabeGabaaabaGaemyzau2aa0baaSqaaiabdMgaPbqaaiabdMha5baakiabcIcaOiabdsha0jabcMcaPiabg2da9GGaciab=z8aMnaaBaaaleaacqWG6bGEaeqaaOGaemyzau2aa0baaSqaaiabdMgaPbqaaiabdMha5baakiabcIcaOiabdsha0jabgkHiTiabigdaXiabcMcaPiabgUcaRiab=H8a5naaBaaaleaacqWG6bGEaeqaaOGaemyzau2aa0baaSqaaiabdMgaPbqaaiabdQha6baakiabcIcaOiabdsha0jabgkHiTiabigdaXiabcMcaPiabgUcaRiab=v7aLnaaDaaaleaacqWGPbqAaeaacqWG5bqEaaGccqGGOaakcqWG0baDcqGGPaqkaeaacqWGLbqzdaqhaaWcbaGaemyAaKgabaGaemOEaOhaaOGaeiikaGIaemiDaqNaeiykaKIaeyypa0Jae8NXdy2aaSbaaSqaaiabdQha6bqabaGccqWGLbqzdaqhaaWcbaGaemyAaKgabaGaemOEaOhaaOGaeiikaGIaemiDaqNaeyOeI0IaeGymaeJaeiykaKIaey4kaSIae8hYdK3aaSbaaSqaaiabdQha6bqabaGccqWGLbqzdaqhaaWcbaGaemyAaKgabaGaemyEaKhaaOGaeiikaGIaemiDaqNaeyOeI0IaeGymaeJaeiykaKIaey4kaSIae8xTdu2aa0baaSqaaiabdMgaPbqaaiabdQha6baakiabcIcaOiabdsha0jabcMcaPaaacaWLjaGaaCzcamaabmaabaGaeG4mamdacaGLOaGaayzkaaaaaa@8812@

where *φ*_*k *_and *ψ*_*k *_are, respectively, the antedependence parameters caused by trait *k *itself and by the other trait, and εiy
 MathType@MTEF@5@5@+=feaafiart1ev1aaatCvAUfKttLearuWrP9MDH5MBPbIqV92AaeXatLxBI9gBaebbnrfifHhDYfgasaacH8akY=wiFfYdH8Gipec8Eeeu0xXdbba9frFj0=OqFfea0dXdd9vqai=hGuQ8kuc9pgc9s8qqaq=dirpe0xb9q8qiLsFr0=vr0=vr0dc8meaabaqaciaacaGaaeqabaqabeGadaaakeaaiiGacqWF1oqzdaqhaaWcbaGaemyAaKgabaGaemyEaKhaaaaa@315D@(*t*) and εiz
 MathType@MTEF@5@5@+=feaafiart1ev1aaatCvAUfKttLearuWrP9MDH5MBPbIqV92AaeXatLxBI9gBaebbnrfifHhDYfgasaacH8akY=wiFfYdH8Gipec8Eeeu0xXdbba9frFj0=OqFfea0dXdd9vqai=hGuQ8kuc9pgc9s8qqaq=dirpe0xb9q8qiLsFr0=vr0=vr0dc8meaabaqaciaacaGaaeqabaqabeGadaaakeaaiiGacqWF1oqzdaqhaaWcbaGaemyAaKgabaGaemOEaOhaaaaa@315F@(*t*) are the time-dependent innovation error terms, assumed to be bivariate normally distributed with mean zero and variance matrix

Σε(t)=(δx2(t)δx(t)δy(t)ρ(t)δx(t)δy(t)ρ(t)δy2(t)),
 MathType@MTEF@5@5@+=feaafiart1ev1aaatCvAUfKttLearuWrP9MDH5MBPbIqV92AaeXatLxBI9gBaebbnrfifHhDYfgasaacH8akY=wiFfYdH8Gipec8Eeeu0xXdbba9frFj0=OqFfea0dXdd9vqai=hGuQ8kuc9pgc9s8qqaq=dirpe0xb9q8qiLsFr0=vr0=vr0dc8meaabaqaciaacaGaaeqabaqabeGadaaakeaaiiqacqWFJoWudaWgaaWcbaacciGae4xTdugabeaakiabcIcaOiabdsha0jabcMcaPiabg2da9maabmaabaqbaeqabiGaaaqaaiab+r7aKnaaDaaaleaacqWG4baEaeaacqaIYaGmaaGccqGGOaakcqWG0baDcqGGPaqkaeaacqGF0oazdaWgaaWcbaGaemiEaGhabeaakiabcIcaOiabdsha0jabcMcaPiab+r7aKnaaBaaaleaacqWG5bqEaeqaaOGaeiikaGIaemiDaqNaeiykaKIae4xWdiNaeiikaGIaemiDaqNaeiykaKcabaGae4hTdq2aaSbaaSqaaiabdIha4bqabaGccqGGOaakcqWG0baDcqGGPaqkcqGF0oazdaWgaaWcbaGaemyEaKhabeaakiabcIcaOiabdsha0jabcMcaPiab+f8aYjabcIcaOiabdsha0jabcMcaPaqaaiab+r7aKnaaDaaaleaacqWG5bqEaeaacqaIYaGmaaGccqGGOaakcqWG0baDcqGGPaqkaaaacaGLOaGaayzkaaGaeiilaWcaaa@6906@

where δx2
 MathType@MTEF@5@5@+=feaafiart1ev1aaatCvAUfKttLearuWrP9MDH5MBPbIqV92AaeXatLxBI9gBaebbnrfifHhDYfgasaacH8akY=wiFfYdH8Gipec8Eeeu0xXdbba9frFj0=OqFfea0dXdd9vqai=hGuQ8kuc9pgc9s8qqaq=dirpe0xb9q8qiLsFr0=vr0=vr0dc8meaabaqaciaacaGaaeqabaqabeGadaaakeaaiiGacqWF0oazdaqhaaWcbaGaemiEaGhabaGaeGOmaidaaaaa@30F0@(*t*) and δy2
 MathType@MTEF@5@5@+=feaafiart1ev1aaatCvAUfKttLearuWrP9MDH5MBPbIqV92AaeXatLxBI9gBaebbnrfifHhDYfgasaacH8akY=wiFfYdH8Gipec8Eeeu0xXdbba9frFj0=OqFfea0dXdd9vqai=hGuQ8kuc9pgc9s8qqaq=dirpe0xb9q8qiLsFr0=vr0=vr0dc8meaabaqaciaacaGaaeqabaqabeGadaaakeaaiiGacqWF0oazdaqhaaWcbaGaemyEaKhabaGaeGOmaidaaaaa@30F2@(*t*) are termed time-dependent innovation variances. These variances can be described by a parametric function such as a polynomial of time [14], but are assumed to be constant in this study. *ρ*(*t*) is the correlation between the error terms of the two traits, specified by an exponential function of time *t *[14], but is assumed to be time-invariant for this study. It is reasonable to say that there is no correlation between the error terms of two traits at different time points, i.e. *Corr*(εiy
 MathType@MTEF@5@5@+=feaafiart1ev1aaatCvAUfKttLearuWrP9MDH5MBPbIqV92AaeXatLxBI9gBaebbnrfifHhDYfgasaacH8akY=wiFfYdH8Gipec8Eeeu0xXdbba9frFj0=OqFfea0dXdd9vqai=hGuQ8kuc9pgc9s8qqaq=dirpe0xb9q8qiLsFr0=vr0=vr0dc8meaabaqaciaacaGaaeqabaqabeGadaaakeaaiiGacqWF1oqzdaqhaaWcbaGaemyAaKgabaGaemyEaKhaaaaa@315D@(*t*_*y*_), εiz
 MathType@MTEF@5@5@+=feaafiart1ev1aaatCvAUfKttLearuWrP9MDH5MBPbIqV92AaeXatLxBI9gBaebbnrfifHhDYfgasaacH8akY=wiFfYdH8Gipec8Eeeu0xXdbba9frFj0=OqFfea0dXdd9vqai=hGuQ8kuc9pgc9s8qqaq=dirpe0xb9q8qiLsFr0=vr0=vr0dc8meaabaqaciaacaGaaeqabaqabeGadaaakeaaiiGacqWF1oqzdaqhaaWcbaGaemyAaKgabaGaemOEaOhaaaaa@315F@(*t*_*z*_)) = 0 (*t*_*y *_≠ *t*_*z*_).

Based on the above conditions, the covariance matrix (**Σ**) of phenotypic values for traits *y *and *z *can be structured in terms of *φ*_*y*_, *φ*_*z*_, *ψ*_*y*_, *ψ*_*z *_and **Σ**_*ε*_(*t*) by a bivariate SAD(1) model [15,16]. Also, the closed forms for the determinant and inverse of **Σ **can be derived as given in [15,16]. We use a vector of parameters arrayed in **Θ**_***v ***_= (*φ*_*y*_, *φ*_*z*_, *ψ*_*y*_, *ψ*_*z*_, *δ*_*y*_, *δ*_*z*_, *ρ*) to model the structure of the covariance matrix involved in the function mapping model.

### Likelihood

The likelihood of samples with 2(*T *1)-dimensional measurements, x=(xi)={yi(t),zi(t)}t=1T
 MathType@MTEF@5@5@+=feaafiart1ev1aaatCvAUfKttLearuWrP9MDH5MBPbIqV92AaeXatLxBI9gBaebbnrfifHhDYfgasaacH8akY=wiFfYdH8Gipec8Eeeu0xXdbba9frFj0=OqFfea0dXdd9vqai=hGuQ8kuc9pgc9s8qqaq=dirpe0xb9q8qiLsFr0=vr0=vr0dc8meaabaqaciaacaGaaeqabaqabeGadaaakeaaieqacqWF4baEcqGH9aqpcqGGOaakcqWF4baEdaWgaaWcbaGaemyAaKgabeaakiabcMcaPiabg2da9iabcUha7jabdMha5naaBaaaleaacqWGPbqAaeqaaOGaeiikaGIaemiDaqNaeiykaKIaeiilaWIaemOEaO3aaSbaaSqaaiabdMgaPbqabaGccqGGOaakcqWG0baDcqGGPaqkcqGG9bqFdaqhaaWcbaGaemiDaqNaeyypa0JaeGymaedabaGaemivaqfaaaaa@49F4@, for individual *i *and marker information, M, in the human population affected by the QTL is formulated on the basis of the mixture model, expressed as

L(ω,Θ|x,M)=∏i=1N[∑i=12ωj|ifj(xi;Θuj,Θv)]     (4)
 MathType@MTEF@5@5@+=feaafiart1ev1aaatCvAUfKttLearuWrP9MDH5MBPbIqV92AaeXatLxBI9gBaebbnrfifHhDYfgasaacH8akY=wiFfYdH8Gipec8Eeeu0xXdbba9frFj0=OqFfea0dXdd9vqai=hGuQ8kuc9pgc9s8qqaq=dirpe0xb9q8qiLsFr0=vr0=vr0dc8meaabaqaciaacaGaaeqabaqabeGadaaakeaacqWGmbatcqGGOaakiiGacqWFjpWDcqGGSaaliiqacqGFyoqucqGG8baFieqacqqF4baEcqGGSaalcqWGnbqtcqGGPaqkcqGH9aqpdaqeWbqaamaadmaabaWaaabCaeaacqWFjpWDdaWgaaWcbaGaemOAaOMaeiiFaWNaemyAaKgabeaakiabdAgaMnaaBaaaleaacqWGQbGAaeqaaOGaeiikaGIae0hEaG3aaSbaaSqaaiabdMgaPbqabaGccqGG7aWocqGFyoqudaWgaaWcbaGaemyDauNaemOAaOgabeaakiabcYcaSiab+H5arnaaBaaaleaacqWG2bGDaeqaaOGaeiykaKcaleaacqWGPbqAcqGH9aqpcqaIXaqmaeaacqaIYaGma0GaeyyeIuoaaOGaay5waiaaw2faaaWcbaGaemyAaKMaeyypa0JaeGymaedabaGaemOta4eaniabg+GivdGccaWLjaGaaCzcamaabmaabaGaeGinaqdacaGLOaGaayzkaaaaaa@63EC@

where the unknown parameters include two parts, *ω *= (*ω*_*j*|*i*_) and **Θ **= (**Θ**_***uj***_, **Θ**_***v***_). In the statistics, the parameters *ω *= (*ω*_*j*|*i*_) determine the proportions of different mixture normals, and actually reflect the segregation of the QTL in the population, which can be inferred on the basis of non-random association between the QTL and the markers. For a mapping population, *N *progeny can be classified into different groups on the basis of known marker genotypes. Thus, in each such marker genotype group, the mixture proportions of QTL genotypes (*ω*_*j*|*i*_) can be expressed as the conditional probability of QTL genotype *j *for subject *i *given its marker genotype.

Suppose that this QTL is genetically associated with a codominant SNP marker that has three genotypes, *MM, Mm *and *mm*. Let *p *and 1 - *p *be the allele frequencies of marker alleles *M *and *m*, respectively, and *D *be the coefficient of (gametic) linkage disequilibrium between the marker and QTL. According to linkage disequilibrium-based mapping theory [17], the detection of significant linkage disequilibrium between the marker and QTL implies that the QTL may be linked with and, therefore, can be genetically manipulated by the marker. The four haplotypes for the marker and QTL are *MA, Ma, mA *and *ma*, with respective frequencies expressed as *p*_11 _= *pq *+ *D*, *p*_10 _= *p*(1 - *q*) - *D*, *p*_01 _= (1 - *p*)*q *- *D *and *p*_00 _= (1 - *p*)(1 - *q*) + *D*. Thus, the population genetic parameters *p*, *q*, *D *can be estimated by solving a group of regular equations if we can estimate the four haplotype frequencies. The conditional probabilities of QTL genotypes given marker genotypes in a natural population can be expressed in terms of the haplotype frequencies (see [18]).

In the mixture model (4), Θ={(Θuj,Θv)}j=02
 MathType@MTEF@5@5@+=feaafiart1ev1aaatCvAUfKttLearuWrP9MDH5MBPbIqV92AaeXatLxBI9gBaebbnrfifHhDYfgasaacH8akY=wiFfYdH8Gipec8Eeeu0xXdbba9frFj0=OqFfea0dXdd9vqai=hGuQ8kuc9pgc9s8qqaq=dirpe0xb9q8qiLsFr0=vr0=vr0dc8meaabaqaciaacaGaaeqabaqabeGadaaakeaaiiqacqWFyoqucqGH9aqpcqGG7bWEcqGGOaakcqWFyoqudaWgaaWcbaGaemyDauNaemOAaOgabeaakiabcYcaSiab=H5arnaaBaaaleaacqWG2bGDaeqaaOGaeiykaKIaeiyFa03aa0baaSqaaiabdQgaQjabg2da9iabicdaWaqaaiabikdaYaaaaaa@40C3@ is the unknown vector that determines the parametric family *f*_*j*_, described by a multivariate normal distribution with the genotype-specific mean vector

uj=(uMj;uPj)={uMj(t);uPj(t)}t=1T−1=[rMj1+(P(t+1)kj)nj−qMjM(t+1);rPjM(t+1−τj)mj−qPjP(t+1)]t=1T−1     (5)
 MathType@MTEF@5@5@+=feaafiart1ev1aaatCvAUfKttLearuWrP9MDH5MBPbIqV92AaeXatLxBI9gBaebbnrfifHhDYfgasaacH8akY=wiFfYdH8Gipec8Eeeu0xXdbba9frFj0=OqFfea0dXdd9vqai=hGuQ8kuc9pgc9s8qqaq=dirpe0xb9q8qiLsFr0=vr0=vr0dc8meaabaqaciaacaGaaeqabaqabeGadaaakeaafaqadeGabaaabaacbeGae8xDau3aaSbaaSqaaiabdQgaQbqabaGccqGH9aqpdaqadaqaaiab=vha1naaBaaaleaacqWGnbqtcqWGQbGAaeqaaOGaei4oaSJae8xDau3aaSbaaSqaaiabdcfaqjabdQgaQbqabaaakiaawIcacaGLPaaacqGH9aqpcqGG7bWEcqWG1bqDdaWgaaWcbaGaemyta0KaemOAaOgabeaakiabcIcaOiabdsha0jabcMcaPiabcUda7iabdwha1naaBaaaleaacqWGqbaucqWGQbGAaeqaaOGaeiikaGIaemiDaqNaeiykaKIaeiyFa03aa0baaSqaaiabdsha0jabg2da9iabigdaXaqaaiabdsfaujabgkHiTiabigdaXaaaaOqaaiabg2da9maadmaabaWaaSaaaeaacqWGYbGCdaWgaaWcbaGaemyta0KaemOAaOgabeaaaOqaaiabigdaXiabgUcaRmaabmaabaWaaSaaaeaacqWGqbaucqGGOaakcqWG0baDcqGHRaWkcqaIXaqmcqGGPaqkaeaacqWGRbWAdaWgaaWcbaGaemOAaOgabeaaaaaakiaawIcacaGLPaaadaahaaWcbeqaaiabd6gaUnaaBaaameaacqWGQbGAaeqaaaaaaaGccqGHsislcqWGXbqCdaWgaaWcbaGaemyta0KaemOAaOgabeaakiabd2eanjabcIcaOiabdsha0jabgUcaRiabigdaXiabcMcaPiabcUda7iabdkhaYnaaBaaaleaacqWGqbaucqWGQbGAaeqaaOGaemyta0KaeiikaGIaemiDaqNaey4kaSIaeGymaeJaeyOeI0ccciGae4hXdq3aaSbaaSqaaiabdQgaQbqabaGccqGGPaqkdaahaaWcbeqaaiabd2gaTnaaBaaameaacqWGQbGAaeqaaaaakiabgkHiTiabdghaXnaaBaaaleaacqWGqbaucqWGQbGAaeqaaOGaemiuaaLaeiikaGIaemiDaqNaey4kaSIaeGymaeJaeiykaKcacaGLBbGaayzxaaWaa0baaSqaaiabdsha0jabg2da9iabigdaXaqaaiabdsfaujabgkHiTiabigdaXaaaaaGccaWLjaGaaCzcamaabmaabaGaeGynaudacaGLOaGaayzkaaaaaa@A035@

and the covariance matrix **Σ**. While the mean vector is determinedby genotype-specific parameters, Θ_*uj *_= (*n*_*j*_, *m*_*j*_, *τ*_*j*_, *r*_*Mj*_, *r*_*Pj*_, *q*_*Mj*_, *q*_*Pj*_, *k*_*j*_), *j *= (2,1,0) the covariance matrix is structured by common parameters, **Θ**_***v ***_= (*φ*_*y*_, *φ*_*z*_, *ψ*_*y*_, *ψ*_*z*_, *δ*_*y*_, *δ*_*z*_, *ρ*).

### Algorithm

Wang and Wu [18] proposed a closed form for the EM algorithm to obtain the maximum likelihood estimates (MLEs) of haplotype frequencies *p*_11_, *p*_10_, *p*_01 _and *p*_00_, and thus the allele frequencies of the marker (*p*) and QTL (*q*) and their linkage disequilibrium (*D*). Genotype-specific mathematical parameters in ***u***_***j ***_(5) for the two differential functions of circadian rhythms, and the parameters that specify the structure of the covariance matrix, **Σ**, can be theoretically estimated by implementing the EM algorithm. But it would be difficult to derive the log-likelihood equations for these parameters by this approach because they are related in a complicated nonlinear way. The simplex algorithm, which relies only upon a target function, has proved powerful for estimating the MLEs of these parameters [19] and will be used in this study. As discussed above, closed forms exist for the determinant and inverse and should be incorporated into the estimation process to increase computational efficiency.

## Hypothesis Testing

One of the most significant advantages of functional mapping is that it can ask and address biologically meaningful questions about the interplay between gene actions and trait dynamics by formulating a series of hypothesis tests. Wu et al. [20] described several general hypothesis tests for different purposes. Although all these general tests can be used directly in this study, we propose here the most important and specific tests for the existence of QTL that affect mRNA and protein changes pleiotropically or separately, and for the effects of the QTL on the shape of differential functions.

### Existence of QTL

Testing whether a specific QTL is associated with the differential functions (1) is a first step toward understanding the genetic architecture of circadian rhythms. The genetic control of the entire rhythmic process can be tested by formulating the following hypotheses:

*H*_0 _: *D *= 0 *vs*. *H*_1 _: *D *≠ 0     (6)

*H*_0 _states that there are no QTL affecting circadian rhythms (the reduced model), whereas *H*_1 _proposes that such QTL do exist (the full model). The statistic for testing these hypotheses (6) is calculated as the log-likelihood ratio (LR) of the reduced to the full model:

*LR*_1 _= -2[ln *L*(Θ˜
 MathType@MTEF@5@5@+=feaafiart1ev1aaatCvAUfKttLearuWrP9MDH5MBPbIqV92AaeXatLxBI9gBaebbnrfifHhDYfgasaacH8akY=wiFfYdH8Gipec8Eeeu0xXdbba9frFj0=OqFfea0dXdd9vqai=hGuQ8kuc9pgc9s8qqaq=dirpe0xb9q8qiLsFr0=vr0=vr0dc8meaabaqaciaacaGaaeqabaqabeGadaaakeaaiiqacuWFyoqugaacaaaa@2E37@, ω˜
 MathType@MTEF@5@5@+=feaafiart1ev1aaatCvAUfKttLearuWrP9MDH5MBPbIqV92AaeXatLxBI9gBaebbnrfifHhDYfgasaacH8akY=wiFfYdH8Gipec8Eeeu0xXdbba9frFj0=OqFfea0dXdd9vqai=hGuQ8kuc9pgc9s8qqaq=dirpe0xb9q8qiLsFr0=vr0=vr0dc8meaabaqaciaacaGaaeqabaqabeGadaaakeaaiiGacuWFjpWDgaacaaaa@2E8F@|**x**, *M*) - ln *L*(Θ^
 MathType@MTEF@5@5@+=feaafiart1ev1aaatCvAUfKttLearuWrP9MDH5MBPbIqV92AaeXatLxBI9gBaebbnrfifHhDYfgasaacH8akY=wiFfYdH8Gipec8Eeeu0xXdbba9frFj0=OqFfea0dXdd9vqai=hGuQ8kuc9pgc9s8qqaq=dirpe0xb9q8qiLsFr0=vr0=vr0dc8meaabaqaciaacaGaaeqabaqabeGadaaakeaaiiqacuWFyoqugaqcaaaa@2E38@, ω^
 MathType@MTEF@5@5@+=feaafiart1ev1aaatCvAUfKttLearuWrP9MDH5MBPbIqV92AaeXatLxBI9gBaebbnrfifHhDYfgasaacH8akY=wiFfYdH8Gipec8Eeeu0xXdbba9frFj0=OqFfea0dXdd9vqai=hGuQ8kuc9pgc9s8qqaq=dirpe0xb9q8qiLsFr0=vr0=vr0dc8meaabaqaciaacaGaaeqabaqabeGadaaakeaaiiGacuWFjpWDgaqcaaaa@2E90@|**x**, *M*)],     (7)

where the tildes and hats denote the MLEs of the unknown parameters under *H*_0 _and *H*_1_, respectively. The LR is asymptotically *χ*^2^-distributed with one degree of freedom.

A similar test for the existence of a QTL can be performed on the basis of these hypotheses, as follows:

*H*_0 _: Θ_*uj *_≡ Θ_*u*_, *j *= (2,1,0)     (8)

*H*_1 _: At least one of the equalities above does not hold;

from which the LR is calculated by

*LR*_2 _: -2[ln *L*(Θ˜˜
 MathType@MTEF@5@5@+=feaafiart1ev1aaatCvAUfKttLearuWrP9MDH5MBPbIqV92AaeXatLxBI9gBaebbnrfifHhDYfgasaacH8akY=wiFfYdH8Gipec8Eeeu0xXdbba9frFj0=OqFfea0dXdd9vqai=hGuQ8kuc9pgc9s8qqaq=dirpe0xb9q8qiLsFr0=vr0=vr0dc8meaabaqaciaacaGaaeqabaqabeGadaaakeaaiiqacuWFyoqugaacgaacaaaa@2E45@|**x**) - ln(Θ^
 MathType@MTEF@5@5@+=feaafiart1ev1aaatCvAUfKttLearuWrP9MDH5MBPbIqV92AaeXatLxBI9gBaebbnrfifHhDYfgasaacH8akY=wiFfYdH8Gipec8Eeeu0xXdbba9frFj0=OqFfea0dXdd9vqai=hGuQ8kuc9pgc9s8qqaq=dirpe0xb9q8qiLsFr0=vr0=vr0dc8meaabaqaciaacaGaaeqabaqabeGadaaakeaaiiqacuWFyoqugaqcaaaa@2E38@, ω^
 MathType@MTEF@5@5@+=feaafiart1ev1aaatCvAUfKttLearuWrP9MDH5MBPbIqV92AaeXatLxBI9gBaebbnrfifHhDYfgasaacH8akY=wiFfYdH8Gipec8Eeeu0xXdbba9frFj0=OqFfea0dXdd9vqai=hGuQ8kuc9pgc9s8qqaq=dirpe0xb9q8qiLsFr0=vr0=vr0dc8meaabaqaciaacaGaaeqabaqabeGadaaakeaaiiGacuWFjpWDgaqcaaaa@2E90@|**x**, *M*)],     (9)

with the doubled tildes denoting the estimates under *H*_0 _of hypothesis (8). It is difficult to determine the distribution of the LR_2 _because the linkage disequilibrium is not identifiable under *H*_1_. An empirical approach to determining the critical threshold is based on permutation tests, as advocated by Churchill and Doerge [21]. By repeatedly shuffling the relationships between marker genotypes and phenotypes, a series of maximum LR_2 _values are calculated, from the distribution of which the critical threshold is determined.

### Is the QTL for mRNA or protein rhythms?

After the existence of a QTL that affects circadian rhythms is confirmed, we need to test whether it affects the rhythmic responses of mRNA and protein jointly or separately. The hypothesis for testing the effect of the QTL on the mRNA response is formulated as

*H*_0 _: (*r*_*Mj*_, *q*_*Mj*_, *k*_*j*_, *n*_*j*_,) ≡ (*r*_*M*_, *q*_*M*_, *k*, *n*)  for* j* = 0, 1, 2    (10)

*H*_1 _: At least one of the equalities above does not hold.

The log-likelihood values under *H*_0 _and *H*_1 _are calculated, and thus the corresponding LR.

A similar test is formulated for detecting the effect of the QTL on the protein rhythm:

*H*_0 _: (*r*_*Pj*_, *q*_*Pj*_, *τ*_*j*_, *m*_*j*_,) ≡ (*r*_*P*_, *q*_*P*_, *τ*, *m*)  for* j* = 0, 1, 2    (11)

*H*_1 _: At least one of the equalities above does not hold.

For both hypotheses (10) and (11), an empirical approach to determining the critical threshold is based on simulation studies. If the null hypotheses of (10) and (11) are both rejected, this means that the QTL exerts a pleiotropic effect on the circadian rhythms of mRNA and protein.

### The QTL responsible for the behavior and shape of circadian rhythms

Two different subspaces of parameters are used to define the features of circadian rhythms: {*n*, *m*, *τ*}, determining the nonlinearity and delay in the system, and {*r*_*M*_, *r*_*P*_, *q*_*M*_, *q*_*P*_}, determining the phase-response curves. The null hypotheses regarding the genetic control of the system's oscillatory behavior and the shape of the rhythmic responses are:

H0:(nj,mj,τj)≡(n,m,τ)H0:(rMj,rPj,qMj,qPj)≡(rM,rP,qM,qP) for j=0, 1, 2(12)
 MathType@MTEF@5@5@+=feaafiart1ev1aaatCvAUfKttLearuWrP9MDH5MBPbIqV92AaeXatLxBI9gBaebbnrfifHhDYfgasaacH8akY=wiFfYdH8Gipec8Eeeu0xXdbba9frFj0=OqFfea0dXdd9vqai=hGuQ8kuc9pgc9s8qqaq=dirpe0xb9q8qiLsFr0=vr0=vr0dc8meaabaqaciaacaGaaeqabaqabeGadaaakeaafaqaaeGabaaabaGaemisaG0aaSbaaSqaaiabicdaWaqabaGccqGG6aGocqGGOaakcqWGUbGBdaWgaaWcbaGaemOAaOgabeaakiabcYcaSiabd2gaTnaaBaaaleaacqWGQbGAaeqaaOGaeiilaWccciGae8hXdq3aaSbaaSqaaiabdQgaQbqabaGccqGGPaqkcqGHHjIUcqGGOaakcqWGUbGBcqGGSaalcqWGTbqBcqGGSaalcqWFepaDcqGGPaqkaeaacqWGibasdaWgaaWcbaGaeGimaadabeaakiabcQda6iabcIcaOiabdkhaYnaaBaaaleaacqWGnbqtdaWgaaadbaGaemOAaOgabeaaaSqabaGccqGGSaalcqWGYbGCdaWgaaWcbaGaemiuaa1aaSbaaWqaaiabdQgaQbqabaaaleqaaOGaeiilaWIaemyCae3aaSbaaSqaaiabd2eannaaBaaameaacqWGQbGAaeqaaaWcbeaakiabcYcaSiabdghaXnaaBaaaleaacqWGqbaudaWgaaadbaGaemOAaOgabeaaaSqabaGccqGGPaqkcqGHHjIUcqGGOaakcqWGYbGCdaWgaaWcbaGaemyta0eabeaakiabcYcaSiabdkhaYnaaBaaaleaacqWGqbauaeqaaOGaeiilaWIaemyCae3aaSbaaSqaaiabd2eanbqabaGccqGGSaalcqWGXbqCdaWgaaWcbaGaemiuaafabeaakiabcMcaPaaacqqGGaaicqqGMbGzcqqGVbWBcqqGYbGCcqqGGaaicqWGQbGAcqGH9aqpcqaIWaamcqGGSaalcqqGGaaicqaIXaqmcqGGSaalcqqGGaaicqaIYaGmcaWLjaWaaeWaaeaacqaIXaqmcqaIYaGmaiaawIcacaGLPaaaaaa@82EC@

The oscillatory behavior of a circadian rhythm can also be determined by the amplitude of the rhythm, defined as the difference between the peak and trough values; its phase, defined as the timing of a reference point in the cycle (e.g. the peak) relative to a fixed event (e.g. beginning of the night phase); and its period, defined as the time interval between phase reference points (e.g. two peaks). The genetic determination of all thesevariables can be tested.

## Simulation

Simulation experiments are performed to examine the statistical properties of the model proposed for genetic mapping of circadian rhythms. We choose 200 individuals at random from a human population at Hardy-Weinberg equilibrium. Consider one of the markers genotyped for all subjects. This marker, with two alleles *M *and *m*, is used to infer a QTL with two alleles *A *and *a *for circadian rhythms on the basis of non-random association. The allele frequencies are assumed to be *p *= 0.6 for allele *M *and *q *= 0.6 for allele *A*. A positive value of linkage disequilibrium *(D *= 0.08) between *M *and *A *is assumed, suggesting that these two more common alleles are in coupled phase [22].

The three QTL genotypes, *AA*, *Aa *and *aa*, are each hypothesized to have different response curves for circadian rhythms of mRNA and protein as described by equation (1). The rhythmic parameters Θ_*uj *_= (*n*_*j*_, *m*_*j*_, *τ*_*j*_, *r*_*Mj*_, *r*_*Pj*_, *q*_*Mj*_, *q*_*Pj*_, *k*_*j*_) for the three genotypes, given in Table 1, are determined in the ranges of empirical estimates of these parameters [10]. Note that for computational simplicity the scaling constant *k *and the total duration of protein production from mRNA are given values 1 and 4.0, respectively. We used the SAD(1) model to structure the covariance matrix based on the antedependence parameters (*φ*_*x*_, *φ*_*y*_, *ψ*_*x*_, *ψ*_*y*_) and innovation variances (δx2
 MathType@MTEF@5@5@+=feaafiart1ev1aaatCvAUfKttLearuWrP9MDH5MBPbIqV92AaeXatLxBI9gBaebbnrfifHhDYfgasaacH8akY=wiFfYdH8Gipec8Eeeu0xXdbba9frFj0=OqFfea0dXdd9vqai=hGuQ8kuc9pgc9s8qqaq=dirpe0xb9q8qiLsFr0=vr0=vr0dc8meaabaqaciaacaGaaeqabaqabeGadaaakeaaiiGacqWF0oazdaqhaaWcbaGaemiEaGhabaGaeGOmaidaaaaa@30F0@, δy2
 MathType@MTEF@5@5@+=feaafiart1ev1aaatCvAUfKttLearuWrP9MDH5MBPbIqV92AaeXatLxBI9gBaebbnrfifHhDYfgasaacH8akY=wiFfYdH8Gipec8Eeeu0xXdbba9frFj0=OqFfea0dXdd9vqai=hGuQ8kuc9pgc9s8qqaq=dirpe0xb9q8qiLsFr0=vr0=vr0dc8meaabaqaciaacaGaaeqabaqabeGadaaakeaaiiGacqWF0oazdaqhaaWcbaGaemyEaKhabaGaeGOmaidaaaaa@30F2@) (Table 1). The innovation variances for each of the two rhythmic traits were determined by adjusting the heritability of the curves to *H*^2 ^= 0.1 and 0.4, respectively, due to the QTL for the rhythmic response at a middle measurement point.

**Table 1:** The MLEs of parameters that define circadian rhythms for three different QTL genotypes, the structure of the covariance matrix and the association between the marker and QTL in a natural population, taking the heritability of the assumed QTL as *H*^2 ^= 0.1. The numbers in parentheses are the square roots of the mean square errors of the MLEs.

Rhythmic Parameters						
	*AA*		*Aa*		*aa*	
	
	Given	MLE	Given	MLE	Given	MLE

*n*	1.50	1.57(0.067)	1.40	1.54(0.139)	1.70	1.74(0.045)
*m*	3.00	3.02(0.018)	2.90	2.91(0.010)	2.80	2.79(0.050)
*r*_*M*_	1.10	1.16(0.060)	1.30	1.35(0.054)	0.90	0.98(0.083)
*r*_*P*_	0.30	0.30(0.020)	0.35	0.36(0.007)	0.40	0.39(0.001)
*q*_*M*_	0.16	0.15(0.008)	0.17	0.16(0.011)	0.18	0.17(0.012)
*q*_*P*_	0.16	0.16(0.001)	0.17	0.16(0.005)	0.18	0.18(0.001)
						
Matrix Structuring Parameters						

	Given MLE					
					
*φ*_*x*_	0.010	0.011(0.001)				
*φ*_*y*_	-0.100	0.098(0.001)				
*ψ*_*x*_	0.100	0.105(0.005)				
*ψ*_*y*_	-0.200	-0.206(0.006)				
δx2 MathType@MTEF@5@5@+=feaafiart1ev1aaatCvAUfKttLearuWrP9MDH5MBPbIqV92AaeXatLxBI9gBaebbnrfifHhDYfgasaacH8akY=wiFfYdH8Gipec8Eeeu0xXdbba9frFj0=OqFfea0dXdd9vqai=hGuQ8kuc9pgc9s8qqaq=dirpe0xb9q8qiLsFr0=vr0=vr0dc8meaabaqaciaacaGaaeqabaqabeGadaaakeaaiiGacqWF0oazdaqhaaWcbaGaemiEaGhabaGaeGOmaidaaaaa@30F0@	0.223	0.223(0.001)				
δy2 MathType@MTEF@5@5@+=feaafiart1ev1aaatCvAUfKttLearuWrP9MDH5MBPbIqV92AaeXatLxBI9gBaebbnrfifHhDYfgasaacH8akY=wiFfYdH8Gipec8Eeeu0xXdbba9frFj0=OqFfea0dXdd9vqai=hGuQ8kuc9pgc9s8qqaq=dirpe0xb9q8qiLsFr0=vr0=vr0dc8meaabaqaciaacaGaaeqabaqabeGadaaakeaaiiGacqWF0oazdaqhaaWcbaGaemyEaKhabaGaeGOmaidaaaaa@30F2@	1.842	1.742(0.100)				
*ρ*	0.200	0.216(0.016)				
						
Genetic Parameters						

	Given MLE					
					
*p*	0.6	0.601(0.003)				
*q*	0.6	0.501(0.094)				
*D*	0.08	0.068(0.012)				

Many factors have been shown to affect the precision of parameter estimation and the power of QTL detection by functional mapping. These factors are related to experimental design (sample size and number and pattern of repeated measures), the genetic properties of the circadian rhythm (heritability of the curves, population genetic parameters of the underlying QTL), and the analytical approach to modeling the structure of the covariance matrix. Previous studies have investigated the properties of functional mapping when different experimental designs are used [15,18]. For this simulation study, we focus on the influence of different heritabilities on parameter estimation using a practically reasonable sample size (*n *= 200). We assumed that the relative concentrations of mRNA and protein are measured at eight equally-spaced time points in each subject, although these measurements can be made differently in terms of the number and pattern of repeated measures.

The phenotypic values of circadian rhythms for the mRNA and protein traits are simulated by summing the genotypic values predicted by the rhythmic curves and residual errors following a multivariate normal distribution, with *MVN*(0, **Σ**). The simulated phenotypic and marker data were analyzed by the proposed model. The population genetic parameters of the QTL can be estimated with reasonably high precision using a closed-form solution approach [18]. We compare the estimation of the marker allele frequencies, QTL allele frequencies and marker-QTL linkage disequilibria under different heritability levels. The precision of estimation of marker allele frequency is not affected by differences in heritability, but estimates of QTL allele frequency and marker-QTL linkage disequilibrium are more precise for a higher (Table 1) than a lower (Table 2) heritability.

**Table 2:** The MLEs of parameters that define circadian rhythms for three different QTL genotypes, the structure of the covariance matrix and the association between the marker and QTL in a natural population, taking the heritability of the assumed QTL as *H*^2 ^= 0.4. The numbers in parentheses are the square roots of the mean square errors of the MLEs.

Rhythmic Parameters						
	*AA*		*Aa*		*aa*	
	
	Given	MLE	Given	MLE	Given	MLE

*n*	1.50	1.52(0.059)	1.40	1.42(0.006)	1.70	1.71(0.060)
*m*	3.00	3.02(0.015)	2.90	2.90(0.009)	2.80	2.80(0.020)
*r*_*M*_	1.10	1.14(0.052)	1.30	1.34(0.028)	0.90	0.93(0.066)
*r*_*P*_	0.30	0.30(0.009)	0.35	0.35(0.002)	0.40	0.40(0.001)
*q*_*M*_	0.16	0.16(0.003)	0.17	0.17(0.011)	0.18	0.18(0.005)
*q*_*P*_	0.16	0.16(0.004)	0.17	0.17(0.003)	0.18	0.18(0.001)
						
Matrix Structuring Parameters						

	Given MLE					
					
*φ*_*x*_	0.010	0.010(0.001)				
*φ*_*y*_	-0.100	0.095(0.002)				
*ψ*_*x*_	0.100	0.102(0.005)				
*ψ*_*y*_	-0.200	0.201(0.006)				
δx2 MathType@MTEF@5@5@+=feaafiart1ev1aaatCvAUfKttLearuWrP9MDH5MBPbIqV92AaeXatLxBI9gBaebbnrfifHhDYfgasaacH8akY=wiFfYdH8Gipec8Eeeu0xXdbba9frFj0=OqFfea0dXdd9vqai=hGuQ8kuc9pgc9s8qqaq=dirpe0xb9q8qiLsFr0=vr0=vr0dc8meaabaqaciaacaGaaeqabaqabeGadaaakeaaiiGacqWF0oazdaqhaaWcbaGaemiEaGhabaGaeGOmaidaaaaa@30F0@	0.307	0.309(0.011)				
δy2 MathType@MTEF@5@5@+=feaafiart1ev1aaatCvAUfKttLearuWrP9MDH5MBPbIqV92AaeXatLxBI9gBaebbnrfifHhDYfgasaacH8akY=wiFfYdH8Gipec8Eeeu0xXdbba9frFj0=OqFfea0dXdd9vqai=hGuQ8kuc9pgc9s8qqaq=dirpe0xb9q8qiLsFr0=vr0=vr0dc8meaabaqaciaacaGaaeqabaqabeGadaaakeaaiiGacqWF0oazdaqhaaWcbaGaemyEaKhabaGaeGOmaidaaaaa@30F2@	0.200	0.204(0.011)				
*ρ*	0.037	0.038(0.002)				
						
Genetic Parameters						

	Given MLE					
					
*p*	0.6	0.601(0.002)				
*q*	0.6	0.67(0.091)				
*D*	0.08	0.067(0.022)				

**Figure 2:**
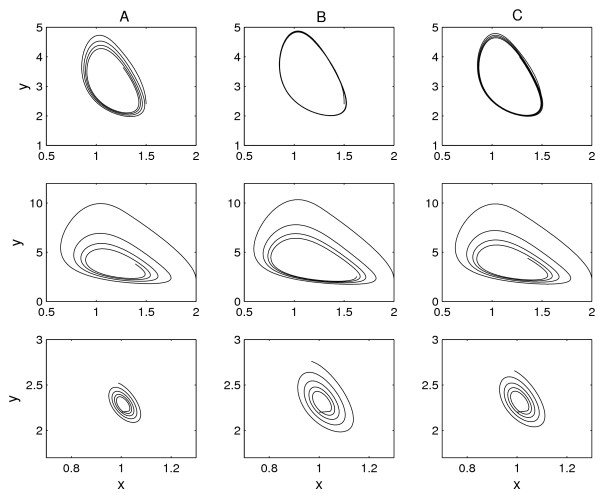
Free-running oscillation of mRNA abundance (*x*) and protein abundance (*y*) in a rhythmic system, expressed as limit cycle contour, annotated with the time points within the 24.6 h circadian cycle, for three assumed QTL genotypes using given rhythmic parameter values (A), estimated values under *H*^2 ^= 0.1 (B), and estimated values under *H*^2 ^= 0.4 (C). The three plots within each column correspond to QTL genotypes *AA*, *Aa *and *aa*, respectively.

Figure 2A illustrates different forms of circadian rhythms for three QTL genotypes, *AA, Aa *and *aa*, with the rhythmic values for the protein and mRNA responses given in Tables 1 and 2. Pronounced differences among the genotypes imply that the QTL may affect the joint rhythmic response of the protein and mRNA concentrations. The rhythmic values can be estimated reasonably from the model. Using the estimates of the rhythmic parameters from one random simulation, we draw the oscillations of the two traits. The shapes of these curves seem to be broadly consistent with those of the hypothesized curves, although the curve estimates are more accurate under higher (Fig. 2C) than lower (Fig. 2B) heritability.

The estimates of the rhythmic parameters for each response curve also display reasonable precision, as assessed by the square roots of the mean square errors over 100 repeated simulations. As expected, the estimate is more precise when the heritability increases from 0.1 (Table 1) to 0.4 (Table 2). The model displays great power in detecting a QTL responsible for circadian rhythms using the marker associated with it. Given the above simulation conditions, a significant QTL can be detected with about 75% power for a heritability of 0.1. The power increases to over 90% as the heritability increases to 0.4.

The model can be used to test whether the QTL detected for overall protein and mRNA rhythm responses also affects key features of circadian rhythms, such as period, amplitude or phase shift, by formulating the corresponding hypotheses. For a real data set, it is exciting to test these hypotheses because they may enable the mechanistic basis of the genetic regulation of circadian rhythms to be identified. In the current simulation, these hypothesis tests were not performed.

## Discussion

One of the most important aspects of life is the rhythmic behavior that is rooted in the many regulatory mechanisms that control the dynamics of living systems. The most common biological rhythms are circadian rhythms, which occur with a period close to 24 h, allowing organisms to adapt to periodic changes in the terrestrial environment [1]. With the rapid accumulation of new data on gene, protein and cellular networks, it is becoming increasingly clear that genes are heavily involved in the cellular regulatory interactions underpinning circadian rhythms [4,23]. However, a detailed picture of the genetic architecture of circadian rhythms has not been obtained, although ongoing projects such as the Human Genome Project will assist in the characterization of circadian genetics.

Traditional strategies for identifying circadian clock genes in mammals have been based on the analysis of single gene mutations and the characterization of genes identified by cross-species homology, and have laid an essential groundwork for circadian genetics [6,23]. However, these strategies do not include a more thorough examination of the breadth and complexity of influences on circadian behavior throughout the entire genome. Genetic mapping relying upon genetic linkage maps has provided a powerful tool for identifying the quantitative trait loci (QTL) responsible for circadian rhythms. In a mapping study of 196 F_2 _hybrid mice, Shimomura et al. [24] detected 14 interacting QTL that contribute to the variation of rhythmic behavior in mice by analyzing different discrete aspects of circadian behavior: free-running circadian period, phase angle of entrainment, amplitude of the circadian rhythm, circadian activity level, and dissociation of rhythmicity.

The data of Shimomura et al. [24] point to promising approaches for genome-wide analysis of rhythmic phenotypes in mammals including humans. Their most significant drawback is the lack of robust statistical inferences about the dynamic genetic control of circadian rhythms. Typically, biological rhythms are dynamic traits, and the pattern of their genetic determination can change dramatically with time. In this article, we have incorporated mathematical models and concepts regarding the molecular and cellular mechanisms of circadian rhythms into a general framework for mapping dynamic traits, called functional mapping [11]. Based firmly on experiments, robust differential equations have been established to provide an essential tool for studying and comprehending the cellular networks for circadian rhythms [1,25-27]. As an attempt to integrate differential equations into functional mapping, the statistical model shows favorable properties in estimating the effects of a putative QTL and its association with polymorphic markers. The simulation study results suggest that the parameters determining the behavior and shape of circadian rhythmic curves can be estimated reasonably even if the QTL effect is small to moderate. As seen in general functional mapping [11], the model implemented with a system of differential equations also allows us to make a number of biologically meaningful hypothesis tests for understanding the genetic control of rhythmic responses in organisms.

As a first attempt of its kind, the model proposed in this article has only considered one QTL associated with circadian rhythms. A one-QTL model is definitely not sufficient to explain the complexity of the genetic control of this trait. A model incorporating multiple QTL and their interactive networks should be derived; this is technically straightforward. In addition, the system of circadian rhythms is characterized by two variables, and this may also be too simple to reflect the complexity of rhythmic behavior. A number of more sophisticated models, governed by systems of five [28], ten [29] or 16 kinetic equations [4,30,31], have been constructed to describe the detailed features of a rhythmic system in regard to responses to various internal and environmental factors. While the identification of circadian clock genes can elucidate the molecular mechanism of the clock, our model will certainly prove its value in elucidating the genetic architecture of circadian rhythms and will probably lead to the detection of the driving forces behind circadian genetics and its relationship to the organism as a whole.

## Competing interests

The author(s) declare that they have no competing interests.

## Authors' contributions

The entire theoretical concept of the work was envisaged by RLW. The mathematical and statistical modeling was carried out by TL with feedback from XLL and YMC.
